# Clinical Potential of Essential Oils: Cytotoxicity, Selectivity Index and Antimicrobial Activity Against Gram-Negative ESKAPEE Pathogens

**DOI:** 10.3390/antibiotics15030274

**Published:** 2026-03-06

**Authors:** Biruk Bayleyegn Belete, Jerome Ozkan, Parthasarathi Kalaiselvan, Muhammad Yasir, Mark Willcox

**Affiliations:** School of Optometry and Vision Science, University of New South Wales, Sydney, NSW 2052, Australiam.willcox@unsw.edu.au (M.W.)

**Keywords:** essential oils, antibiotics, ESKAPEE pathogens, selectivity index, cytotoxicity

## Abstract

**Background:** Novel therapeutic compounds with strong efficacy and low resistance potential are urgently needed to combat life-threatening infections caused by antibiotic-resistant ESKAPEE pathogens. These pathogens contribute globally to a large share of bloodstream, respiratory, urinary, and wound infections, and often have levels of high antimicrobial resistance. This review examined the antimicrobial efficacy of different plant essential oils (EOs) against Gram-negative ESKAPEE pathogens and their cytotoxic effects and calculated selectivity indices in cancer and normal cell lines. **Methods**: This review was developed using studies retrieved from PubMed, Scopus, and Web of Science, covering publications between 2013 and 2024 using the search terms: “essential oils”, “plant extracts”, “safety”, “cytotoxicity”, “cell lines”, “human”, “in-vitro”, antimicrobial”, “antibacterial” and “antibiotic” with Boolean operators (“AND”, “OR”, “NOT”). Only studies that reported both antimicrobial inhibitory concentrations and concentrations causing toxicity to mammalian cells were included in the final review. These data were then used to calculate the selectivity indices of the EOs (toxic concentration/antimicrobial inhibitory concentration) to give an initial assessment of safety. **Results:** *Ocimum basilicum* EOs had strong antibacterial effects against the Gram-negative ESKAPEE pathogens *Escherichia coli*, *Pseudomonas aeruginosa*, and *Klebsiella pneumoniae*, with minimum inhibitory concentrations (MICs) as low as 1 μg/mL and high selectivity indices of >80.4. Likewise, *Satureja nabateorum* EOs had potent antibacterial activity, with a low MIC of 0.1 μg/mL against *K. pneumoniae*, 2.3 μg/mL against *E. coli*, and 12.5 μg/mL against *P. aeruginosa*, along with a very high selectivity index (>100). Other EOs such as those from *Eucalyptus* spp., *Thymus* spp., *Mentha* spp., *Cinnamomum* spp., *Artemisia* spp., and *Aquilaria crassna* also had broad-spectrum antibacterial potential and minimal toxicity toward mammalian cells, making them promising candidates for safe and effective antimicrobial agents in clinical and industrial applications. However, several EOs had selectivity indices of <10, indicating that at their MIC they would also be potentially highly cytotoxic. EOs tended to show increased toxicity to cells derived from cancers. **Conclusions and recommendations:** Certain EOs are highly active against Gram-negative ESKAPEE pathogens. They are also toxic to cancer-derived mammalian cells. Additional studies using normal cell lines and clinical trials are warranted to further validate their safety and therapeutic potential.

## 1. Introduction

Plants produce EOs as a part of their secondary metabolism, where they play a key role in plant defense or mediating the interactions of plants with the environment. They are usually in low concentrations but can be extracted from various structures, including bark, leaves, flowers, buds, seeds, roots, and fruits, and concentrated [1]. A single EO can contain ≥400 different compounds depending on the extraction method employed, with the majority of compounds present at concentrations of less than 1% [2].

Due to the emergence of new and severe diseases such as cancer, anxiety, obesity, bronchial asthma, aged related conditions and immunodeficiency syndromes, the use of herbal medicines has recently increased in industrialized countries [3]. In developed countries, approximately 10–50% of people regularly use herbal medicines, often valued for their perceived immune-boosting effects. They can be some of the most accessible and affordable treatments for common conditions such as colds, gastrointestinal issues, and joint pain [4]. However, several challenges hinder their clinical translation, including variability in plant material quality, absence of standardized dosing, risk of contamination, inadequate regulatory oversight, limited support from healthcare professionals, and a scarcity of well-designed scientific studies [3,5].

**Figure 1:**
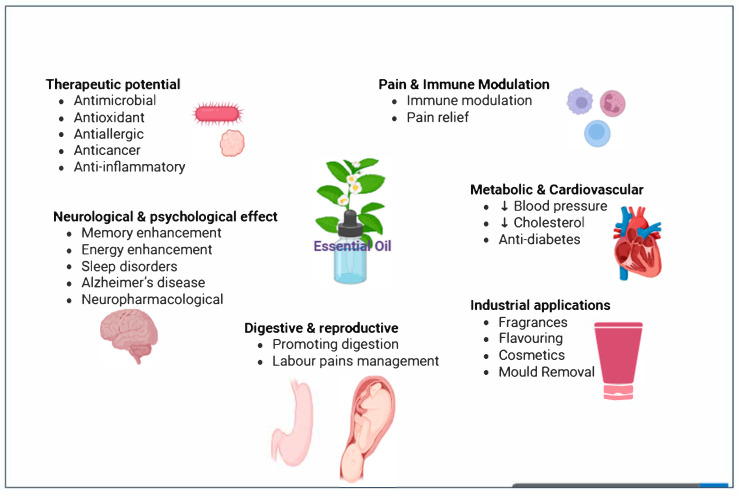
Overview of EO applications in health and industries [6,7,8,9,10,11,12,13,14]. Therapeutic potential: Some EOs in topical formulations are used for wound healing, medicated shampoos and scrubs for the treatment of fungal and bacterial infections, and preparations used for conditions such as acne and asthma. Pain and immune modulation: Oils such as frankincense and others have been reported to exert immunomodulatory effects. EOs are also commonly used for pain relief, particularly through massage applications. Neurological and psychological effects: Some EOs also play important roles in neurological and psychological effects, particularly when administered through inhalation or vapor exposure. Metabolic and cardiovascular: EOs, such as cinnamon, have been reported to exhibit antidiabetic effects by enhancing insulin secretion or improving insulin activity. Digestive and reproduction: Several EOs are currently under investigation for their ability to promote relaxation and emotional comfort during labor. Industrial applications: EOs are used as flavorings, such as peppermint in chewing gum, and EOs are used to reduce mold build up in air-conditioning units. As EOs have gained immense popularity in recent years, the global market for these products has also seen significant growth. An analysis of global trade in medicinal and aromatic plants from 2010 to 2023 [4] found that China and India were the primary exporters, while the United States, Germany, and Japan were the main importers. Down arrows represent decreases in the relevant criteria.

EOs are rich complex mixtures of therapeutic molecules, with volatile terpenes and terpenoids often being their principal bioactive compounds. Due to these bioactive compounds, EOs can have a wide range of biological and pharmacological activities as well as industrial applications, as illustrated in Figure 1.

### Gram-Negative ESKAPEE Pathogens

The growing prevalence of antibiotic resistance and the declining development of new antimicrobials, particularly to combat ESKAPEE pathogens (*Enterococcus faecium*, *Staphylococcus aureus*, *Klebsiella pneumoniae*, *Acinetobacter baumannii*, *Pseudomonas aeruginosa*, *Enterobacter* sp. and *Escherichia coli*), have been identified as major emerging threats to global public health [15]. According to the World Health Organization (WHO) 2025 surveillance report, the rising trend of antibiotic resistance among Gram-negative bacterial pathogens within ESKAPEE pathogens represents an escalating global threat [16]. This resistance is linked to severe clinical consequences and a lack of effective treatment options, particularly in low-income countries, which increases the urgent demand for novel and fast-acting alternatives.

ESKAPEE bacteria are high-priority pathogens because of high morbidity, mortality and rapidly evolving resistance to last-line antibiotics such as carbapenems and polymyxins [17]. Within Gram-negative ESKAPEE pathogens, carbapenem-resistant *K pneumoniae* has been identified as the top-ranked pathogen because of its high resistance levels, association with hospital-acquired infections, and limited treatment options. *K. pneumoniae*’s multi-drug resistance (MDR) nature and high mortality rates, especially among immunocompromised individuals, make it a serious threat capable of causing a wide range of infections, including abscesses, respiratory and urinary tract infections, intestinal and soft tissue infections, and sepsis [18].

According to the three-tier WHO priority classification (critical, high, and medium), carbapenem-resistant *A. baumannii* and carbapenem- and third-generation cephalosporin-resistant Enterobacterales (that include *E. coli*) are classified under the critical priority tier, while carbapenem-resistant *P. aeruginosa* is placed in the high-priority category [19]. *A. baumannii* has emerged as a major global health concern in hospital environments, with extensive resistance to commonly used antibiotics through MDR, extensively drug-resistant (XDR), and pandrug-resistant (PDR) strains [20]. *P. aeruginosa* is an opportunistic pathogen with multiple inherent resistance mechanisms, including reduced drug permeability and a diverse set of multidrug efflux pumps [21]. Although *E. coli* was only recently incorporated into the expanded ESKAPEE group, its escalating AMR and frequent involvement in hospital-acquired infections have positioned *E. coli* as a major and rising threat to global public health [22].

Due to the prevalence of MDR in the ESKAPEE group, new antimicrobial agents are urgently needed. The antibacterial activities of various EOs against Gram-positive ESKAPE pathogens, along with their safety and selectivity indices (SI), have recently been described [23]. In addition, EOs from species such as *Melaleuca alternifolia* (tea tree), *Thymus vulgaris* (thyme), *Ocimum basilicum* (basil), *Eucalyptus* spp., *Lavandula* spp. (lavender), and clove can be inhibitory against a range of Gram-negative bacteria, including *P. aeruginosa*, *E. coli*, *Acinetobacter* spp., and *K. pneumoniae* [24,25,26].

EOs and their derivatives can overcome bacterial defenses by disrupting cell membranes, coagulating cytoplasmic contents, inhibiting efflux pumps, preventing biofilm formation, interfering with metabolic pathways, inducing oxidative stress, and ultimately causing cell lysis [23]. They can exert antibacterial activity against Gram-negative bacteria through several complementary mechanisms. Many EO components, such as carvacrol or thymol, disrupt the integrity and permeability of bacterial cell membranes, leading to leakage of ions and metabolites and ultimately cell death [27,28]. EOs also interfere with key resistance-associated pathways, including inhibition of efflux pumps, disruption of quorum-sensing (QS) communication [29], and suppression of biofilm formation [30]. In addition, some EOs enhance antibiotic susceptibility by increasing membrane permeability and synergizing with conventional antibiotics. For example, carvacrol has been shown to potentiate tobramycin activity via enhanced membrane disruption [27]. Collectively, these mechanisms highlight the multifaceted antimicrobial potential of EOs against Gram-negative bacteria and support their relevance as promising adjunct or alternative therapeutic agents.

Although many spices and their major constituents are listed as Generally Recognized as Safe (GRAS) by the United States Food and Drug Administration (FDA) for use as flavoring agents and food preservatives [13], their safety profile in therapeutic applications remains a subject of active investigation. A recent comprehensive study on the antimicrobial and cytotoxic properties of various EOs and their derivatives demonstrated that EOs derived from *Eucalyptus* spp., *Cinnamomum*, *Mentha*, *Thymus*, *Syzygium aromaticum*, and *Stachys parviflora*, among others, exhibit strong antibacterial activity against Gram-positive ESKAPE pathogens [23]. Notably, these EOs showed no significant cytotoxic effects on cell lines. These findings highlight the promising potential of these natural compounds for further development as safe and effective therapeutic agents against Gram-positive ESKAPE pathogens.

The aims of this review were to evaluate the antimicrobial efficacy, cytotoxicity and safety profiles of EOs in reducing the spread of Gram-negative ESKAPEE pathogens. A novel aspect of this review is the inclusion of calculated SI values, which provide critical insight into the therapeutic potential and clinical applicability of EOs against infectious diseases. Furthermore, the review explores a broad range of EOs derived from food spices and traditional medicinal sources that are currently available in the market, highlighting their potential role in combating MDR pathogens.

## 2. Results and Discussion

**Figure 2:**
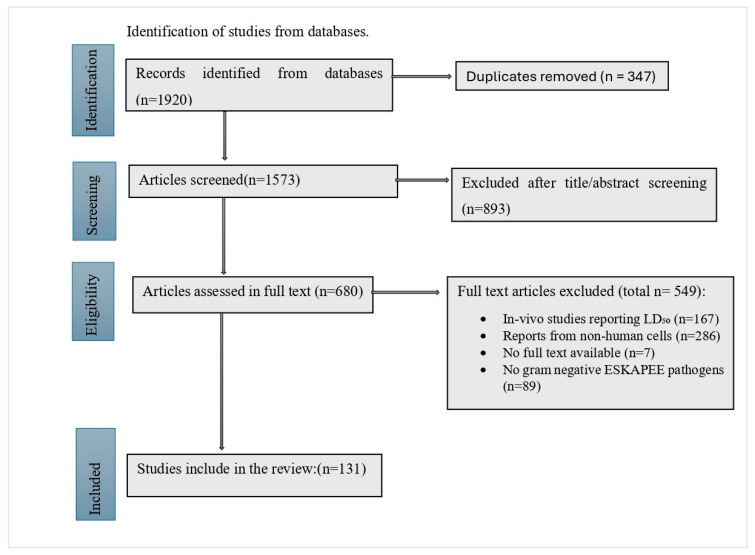
Flowchart of database search and study selection for evaluating the antibacterial and cytotoxic effects of various EOs.

Study selection:

A total of 1920 records were identified through database searches. After removal of duplicates, 1573 articles were screened by title and abstract, of which 680 underwent full-text assessment. Following full-text review, 549 articles were excluded for several reasons, including reporting MIC data without corresponding cytotoxicity results (or vice versa), as well as studies conducted using non-mammalian cell models, which limited direct comparison with human cell data. Ultimately, 131 studies were included in the final review for evaluating the effectiveness, cytotoxicity, and safety of EOs against Gram-negative ESKAPEE pathogens (Figure 2).

Most of the studies included in this review employed American Type Culture Collection (ATCC) reference strains for antimicrobial testing. Two studies [31,32] employed the Microbial Type Culture Collection (MTCC), one study [33] used the Agricultural Culture Collection of China (ACCC) and three studies [34,35,36] used the Persian Type Culture Collection (PTCC). In contrast, only a limited number of studies (n = 3) evaluated clinical isolates or MDR strains [37,38,39].

1. Efficacy and safety of EOs against *K. pneumoniae* and *A. baumannii*.

As shown in Table 1, several EOs had strong antibacterial activity against *K. pneumoniae* while having minimal cytotoxicity toward tested mammalian cells, indicating selective toxicity against bacterial cells over mammalian cells. For instance, *S. nabateorum* showed an exceptionally low MIC of 0.1 µg/mL against *K. pneumoniae* with a high maximum SI of 8074.1 [40]. as Additionally, *Artemisia* sp. had MICs of 2.5–25 µg/mL and an SI of 6164 [41,42], reflecting a very wide safety margin. Similarly, *Thymus* sp. had MICs of 1.6–12.5 µg/mL and an SI of 86.8 [38], and *Ocimum basilicum* had an MIC of 1 µg/mL with a maximum SI of 80.4 [43].

In addition to its strong antibacterial activity against *K. pneumoniae*, *S. nabateorum* had good effectiveness against other clinically important Gram-negative pathogens. It demonstrated substantial inhibitory activity against *P. aeruginosa*, with a reported MIC of 12.5 μg/mL (Table 2). Even more notably, this EO exhibited pronounced potency against *E. coli*, achieving an MIC as low as 2.3 μg/mL (Table 3). These findings highlight the broad-spectrum antibacterial potential of *S. nabateorum* as well as its potential safety, underscoring its value as a promising natural antimicrobial candidate. These findings align with previous studies that have reported potent antibacterial activities and a favorable SI for *Satureja intermedia* [44,45].

Studies on *Thymus vulgaris* EOs have shown its broad-spectrum antibacterial activity and low cytotoxicity, reinforcing its potential for clinical applications [46]. *Ocimum basilicum, S. nabateorum* and *Thymus vulgaris* EOs not only had good antimicrobial potency against Gram-negative ESKAPE pathogens but also show potential antibacterial activity against Gram-positive ESKAPE pathogens, including *S. aureus*, *E. faecium*, and even methicillin-resistant *S. aureus* (MRSA) strains [23]. These results highlight that these EOs possess potent antibacterial effects while maintaining safety toward mammalian cells, making them promising candidates for further in vivo studies and potential clinical applications against both Gram-positive and Gram-negative ESKAPE pathogens.

However, some EOs, including those from *Citrus* sp. (SI_max_ = 0.01) [47], *Diospyros discolor* (SI_max_ = 0.3) [48], *Momordica charantia* (SI_max_ = 0.04) [37], Rose oil (SI_max_ = 0.1) [49], and others, had concentrations required to inhibit *K. pneumoniae* higher than those that killed mammalian cells, indicating toxicity risks. This indicates that the concentration required to effectively inhibit bacterial growth may also cause adverse effects on mammalian cells. In addition to the in vitro evidence, a clinical trial demonstrated that *Citrus reticulata* (tangerine; 86% d-limonene) EO exhibited dose-dependent cytotoxicity and significantly enhanced skin penetration when applied at higher concentrations compared with *Melaleuca* and lavender oils [50]. Consequently, the interpretation of SI values derived from in vitro studies for clinical safety and efficacy is influenced by methodological heterogeneity, including differences in MIC determination (microbial strains, inoculum size, growth media, incubation time, and endpoint definition), heterogeneity in cytotoxicity assays (cell type, assay platforms, exposure duration, and viability thresholds), and different solvents and solvent concentrations [51,52]. Clinical studies provide more relevant evidence of EO safety than in vitro models alone.

The chemical constituents of EOs are the primary determinants of their biological and pharmacological activities, and the composition of each EO is summarized in each table. However, even EOs derived from the same plant species can differ between studies due to regional variations, differences in plant parts used, seasonal and environmental factors, and the extraction methods employed. Only a few studies have simultaneously assessed the antimicrobial effects of EOs against *A. baumannii* or *Enterobacter* spp. alongside their cytotoxicity profiles. *Artemisia turanica* had high safety toward normal lymphocyte cells (IC_50_ = 3291.49 μg/mL) compared to cancerous HeLa cancer cells (IC_50_ = 17.7 μg/mL), resulting in a maximum SI of 285.6 [53], suggesting its potential as a safe natural antibacterial agent (Table 1). In contrast, *Momordica charantia*, *Pimenta dioica*, and *Rosmarinus officinalis* had MICs against *A. baumannii* exceeding the IC_50_ of the tested cells, indicating that these oils may not be safe for future antibacterial applications, at least without further formulations to reduce toxicity but maintain efficacy.

**Table 1:** Summary of mammalian cell inhibitory concentration (IC_50_), MIC and corresponding SI values for EOs (arranged alphabetically, A–Z) tested against *K. pneumoniae* and *A. baumannii*.

Pathogens	Plant Name or EO	Major Chemical Constituent (Amount or Relative Amount)	Cell Lines Applied in Toxicity Test	Mammalian Cell Inhibitory Concentration (IC_50_) μg/mL	MIC (μg/mL)	SI_min_	SI_max_	References
*K. pneumonia*	*Achillea coarctata*	camphor (29%)	**NIH/3T3**, MCF7	37.9–42.3	250	0.15	0.17 ^d^	[54]
	*Aloysia citriodora*	geranial (α-citral) (47.6%)	HeLa	47	5	9.4	9.4 ^c^	[55]
	*Aquilaria crassna*	β-caryophyllene (not given)	**CCD-18Co**, **NIH/3T3-L1**, MCF-7, K562, PC3, ME-180, HT-29, PANC-1, HCT 116	19–530	14	1.4	43.7 ^b^	[31]
	*Artemisia* sp.	β-thujone (89.54%).	HeLa, MCF-7, HepG2, Caco-2	326–15,412	2.5–25	13.1	6164 ^a^	[41,42]
	*Citrus* sp.	limonene (43.2%)	MCF-7, Caco-2, HepG2, **LX-2**, HeLa	338–534	50,000	0.006	0.01 ^d^	[47]
	*Cousinia* sp.	*m*-benzyl benzyl alcohol (46.7%)	T-47D, A549, Hep-G2, A2780	4.5–32.2	62.5–125	0.04	0.5 ^d^	[56]
	*Diospyros discolor*	(2Z,6E)-farnesol	J5, A549, HT-29	10.6–38.6	125	0.08	0.29 ^d^	[48]
	*Ephedra intermedia*	2-ethyl-pyrazine (67.37%)	LnCap, HCT119, Hela	23.2–616.3	500	0.05	1.23 ^c^	[57]
	*Ferulago trifida Boiss*	suberosin (20.7%)	HT-29, A-549, MCF-7	24–40	125–250	0.1	0.4 ^d^	[58]
	*Illicium verum*	E-anethole (88.4%)	MCF-7, 3T3, HeLa, **LX-2**	57.3–131.7	100	0.6	1.3 ^c^	[59]
	*Iris haynei*	diethyl phthalate (65.8%)	CaCo-2, HepG2, B16F1	757.9–915.5	25	30.3	36.6 ^b^	[60]
	*Laurus nobilis*	1,8-cineole (48.54%)	B16F1, MCF-7, CaCo-2	99.1–324	50	2	2.6 ^c^	[61]
	*Mentha* sp.	carvone (57.7–65.6%)	MCF-7, **HUVEC**, A 2780, A549	36–214	40–70	0.61	5.2 ^c^	[62]
	*Momordica charantia*	quercetin-O-pentosylhexoside	Hep2G, MCF-7, NCI-H467, HeLa,	112–210	500	0.02	0.04 ^d^	[37]
	*Nepeta* sp.	trans-caryophyllene (17.53%)	**T lymphocytes**, C450, KB, A549	133–5267.4	2500	0.1	2.1 ^c^	[63]
	*Ocimum basilicum*	linalool (not given)	MCF-7, Hep3B, HeLa	53.7–80.4	1	53.7	80.4 ^b^	[43]
	*Pistacia lentiscus*	limonene (43.78%)	HeLa	169	25	6.8	6.8 ^c^	[64]
	Rose oil	citronellol (36%)	**Beas-2B**, A549	16.6–85.6	750	0.02	0.1 ^d^	[49]
	*Satureja nabateorum*	thymol (43%)	MCF-7, COLO-205, HepG2, HeLa,	82–1090	0.14	607.4	8074.1 ^a^	[40]
	*Scorzonera calyculata* Boiss	6,10,14-trimethyl-2-pentadecanone (27.73%)	A549	9.8	200	0.05	0.05 ^d^	[65]
	*Tetraclinis articulata*	α-pinene (36.5%)	RAW 264.7	577.32	1000	0.58	0.58 ^d^	[66]
	*Thymus* sp.	carvacrol (45.53%) [32], p-cymene (29.52%) [38]	HeLa, A375, HepG-2, PC-3, LS 174 T	0.21–135	1.6–12.5	0.02	86.8 ^b^	[32,38]
	*Withania adpressa* Coss	caryophyllene (24.74%)	**MCF-12**	1000	46	21.7	21.7 ^b^	[67]
	*Xylopia aethiopica*	pinocarvone (26.5%)	RAW 264.10	3.8	32	0.12	0.12 ^d^	[68]
*A. baumannii*	*Artemisia* sp.	1,8-cineole (32–35%)	HeLa, **lymphocytes**	7.1–6322.7	20–5000	0	285.6 ^a^	[53,69,70,71]
	*Momordica charantia*	quercetin-O-pentosylhexoside	HeLa, Hep2G, MCF-7, NCI-H464	112–210	10,000	0.01	0.02 ^d^	[37]
	*Pimenta dioica*	eugenol (90%)	THP-1,	29.6	500	0.06	0.06 ^d^	[72]
	*Rosmarinus officinalis*	*α*-pinene (27%)	THP-1	14.2	500	0.03	0.03 ^d^	[72]

**Bold:** normal cell lines; normal font: cancerous cell lines; SI_min_: minimum selectivity index; SI_max_: maximum selectivity index. (^a^) = maximum selectivity index > 100; (^b^) = maximum selectivity index 10–99; (^c^) = maximum selectivity index 1–9; (^d^) = maximum selectivity index < 1. Full names of cells are provided in the Supplementary Materials.

2. Efficacy and safety of EOs against *P. aeruginosa*.

The data represented in Table 2 show that EOs derived from *Eucalyptus cinerea* (MIC 0.4 μg/mL; SI 1658.2) [73], *Artemisia* sp. (SI: 5711.7) [74], *S. nabateorum* (MIC 12.5 μg/mL; SI: 148.8) [40], *Ocimum basilicum* (MIC 1 μg/mL; SI: 80.4) [43], and others, demonstrated remarkable antibacterial activity against *P. aeruginosa* while maintaining low cytotoxicity across a range of cell lines.

*Eucalyptus cinerea* is among the Eucalyptus species with the highest reported concentrations of 1,8-cineole (also called eucalyptol), and the presence of this compound may explain the species’ antibacterial activity, as it can increase membrane permeability, dissipate the proton motive force, cause leakage of cellular contents and prevent bacterial cell growth [75]. The activity of *Eucalyptus* oil has been shown to be concentration-dependent [76]. It has the potential to be used as an alternative drug in treatment as an external ointment for wound infection by *P. aeruginosa* and perhaps for other *P. aeruginosa*-related infections, including scarlet fever, upper respiratory tract infections, and food poisoning [76,77].

*Artemisia* sp. is a widely distributed genus found across the globe, except in Antarctica [78], and it exhibits a broad spectrum of pharmacological activities, including antibacterial, antiulcer, anticancer, hepatoprotective, antidiabetic, antioxidant, anti-inflammatory, antiepileptic, and antimalarial effects, as well as therapeutic potential against asthma, gastritis, coughs, colds and fever [79]. EOs from *Artemisia* sp. exhibit broad-spectrum antibacterial activity through multiple mechanisms, including the induction of potassium and phosphate ion leakage, disruption of the cell wall, and alterations in cell morphology leading to cell shrinkage [80,81]. These effects are primarily attributed to major constituents such as 1,8-cineole and α/β-pinene [82].

**Table 2:** Mammalian cell inhibitory concentration (IC_50_), MIC and corresponding SI values for EOs (arranged alphabetically, A–Z) tested against *P. aeruginosa* and *Enterobacter* sp.

Pathogens	Plant Name or EO	Major Chemical Constituent (Amount or Relative Amount)	Cell Lines Used in Toxicity Test	Mammalian Cell Inhibitory Concentration (IC_50_) μg/mL	MIC (μg/mL)	SI_min_	SI_max_	References
*P. aeruginosa*	*Actinidia arguta*	squalene (23.08%)	A549, HT-29, PC-3	6.1–13.7	1.6	3.8	8.8 ^c^	[33]
	*Aeschynomene indica*	(*E*)-caryophyllene (17.3%)	HepG2, MCF-7, **LO2**	40.8–74.1	1.3–2.5	20.5	48.3 ^b^	[83]
	*Allium hooshidaryae*	menthol (19.0%)	MCF-10, MOLT-4	109.2–297.5	62.5	1.7	4.8 ^c^	[84]
	*Aniba parviflora*	leaves: β-Phellandrene (15.1%), branches: γ-Eudesmol (16.8%)	MCF-7	67.9	1250	0.05	0.05 ^d^	[85]
	*Aquilaria crassna*	β-caryophyllene (not given)	**CCD-18Co**, HCT 116, HT-29, K562, MCF-7, ME-180, **NIH/3T3**-L1, PANC-1, PC3, RGC5	27–612	7	3.9	87.4 ^b^	[31]
	*Artemisia* sp.	β-thujone (89.54%) [41], camphor (30.21%) [70], 1,8-cineole (35.2%) [71]	HeLa, **lymphocytes**	7.1–5711.7	1–5000	0	5711.7 ^a^	[41,70,71]
	*Asteraceae caerulescens*	davana ether (17.3%)	A375, HCT116, MDA-MB 231	5.2–7.6	6	0.9	1.3 ^c^	[74]
	*Bocageopsis multiflor*	cis-linalooloxide (furanoid) (33.1%)	**L929**	1.4	4.7	0	0 ^d^	[86]
	*Buxus macowanii*	neophytadiene (90%)	**WI-38**	20.4	2500	0	0 ^d^	[87]
	*Centaurea irritans* Wagenitz	geranial (38.62%)	MCF-7	10.3	100	0.05	0.05 ^d^	[65]
	*Cinnamomum*	eucalyptol (65.87%) [88], (E)-cinnamaldehyde (71.50%) [89]	HCT-116, HepG2, MCF-7, **adipose-derived mesenchymal stem cells**	9.1–83,510	3.1–3125	1.2	26.7 ^b^	[88,89]
	*Cistus* sp.	α-pinene (42.1%)	**NIH-3T3**, PC-3, MCF-7, SH-SY5Y, AGS, CaCo2, NCI-H460, PLP2,	13.9–207	256–2500	0.01	0.1 ^d^	[39,90]
	*Citrus* sp.	D-limonene (77.6%) [91], limonene (37.5%) [92]	A549, FemX, HeLa, K562, **MRC-5**, HepG2, **LX-2**, Caco-2, MCF-7, MDAMB231	25.7–534	50–50,000	0.01	2 ^c^	[91,92,93]
	*Cousinia* sp.	*m*-benzyl benzyl alcohol (46.7%)	A2780, A549, Hep-G2, T-47D	4.5–32.2	31.3–62.5	0.07	1 ^c^	[56]
	*Cuminum cyminum*	cumin aldehyde (19.54)	A2780, DU-145 , MCF-7, PC3, U-87-MG	22–41.1	320	0.07	0.1 ^d^	[34]
	*Curcuma* sp. (Turmeric)	ar-turmerone (21.67%)	HepG2, LNCaP, melanoma B16	4.6–429	391–706.4	0.01	0.7 ^d^	[94,95]
	*Cymbopogon martiniivar*	geraniol (76.9%)	PLP2, HCT-15, HeLa, HepG2, MCF-7, NCI-H460	39.2–358	250–500	0.1	1.4 ^c^	[96]
	*Diospyros discolor*	(2Z,6E)-farnesol	A549, HT-29, J5	10.6–36.8	250	0.04	0.2 ^d^	[48]
	*Dracocephalum kotschyi* Boiss	geranial (citral a) (12.1%)	HeLa, **lymphocytes**	26.4–4266.7	160–320	0.08	26.7 ^b^	[97]
	*Ephedra intermedia*	2-ethyl-pyrazine (67.4%)	Hela, LnCap	23.2–616.2	330	0.07	1.9 ^c^	[57]
	*Eucalyptus cinerea*	1,8-cineole (55.24%)	Calu-3, Jurkat	391.4–689.8	0.4	940	1658.2 ^a^	[73]
	*Eucalyptus globulus*	1,8-cineol (74.3%)	A2780, C-26, DU-145, Hela, MCF-7, PC3	33.2–51.4	112	0.3	0.5 ^d^	[98]
	*Ferula* sp.	δ-3-carene (72.6%) [99], caryophyllene oxide (33.9%) [100]	**L929**, HeLa, HepG-2, HCT-119, HT-31, HT-29, HCT-116, A549, U937	3.4–252.2	78–1250	0.04	1 ^c^	[99,100]
	*Ficus tikoua*	palmitic acid (51.13%)	NCI-H1299, **MRC-5**, K565, A549	31.7–161.1	6250	0.01	0.03 ^d^	[101]
	*Gelsemium elegans*	α-terpineol (18.8%)	HT-29, HCT-116, HeLa, A549, U937	3.4–46.7	78	0.04	0.3 ^d^	
	*Hedychium* sp.	coronarin E (20.3%) [102], linalool (26.5%) [103]	A549, K562, **L929**, **MRC-5**, NCI-H1299, PC-3, L933, RAW264.11	0.4–7074	6.5–312.5	0.09	22.6 ^b^	[102,103]
	*Illicium verum*	E-anethole (88.4%)	**LX-2**, 3T3, HeLa, MCF-7	57.3–131.7	100	0.6	1.3 ^c^	[59]
	*Iris haynei*	diethyl phthalate (65.8%)	CaCo-2, HepG2, B16F10	757.9–915.5	66.6	11.5	13.7 ^b^	[60]
	*Laurus nobilis*	1,8-cineole (48.54%)	MCF-7, CaCo-2, B16F10	99.1–324.1	50	2	6.5 ^c^	[61]
	*Leptospermum petersonii*	geranial (32.9%)	HCT-15, HeLa, HepG2, MCF-7, NCI-H460	5.6–316.8	250	0.02	1.3 ^c^	[96]
	*Limonium oleifolium*	γ-muurolene (10.8%)	J 776	90.2	2	45.1	45.1 ^b^	[104]
	*Melaleuca alternifoila*	terpinen-4-ol (38.6%)	A549, FemX, HeLa, K562, LS-174, **MRC-5**	48.8–70.5	630	0.08	0.1 ^d^	[92]
	*Mentha* sp.	menthol (33.5%)	**NIH 3T3**, A552, HeLa, LS-177, **MRC-8**	92–382	20–24.9	4.6	15.4 ^b^	[105]
	*Mikania micrantha*	isoledene (16%)	Hela, L6, MIAPaCa2, PA1	5.4–82.2	16	0.3	5.1 ^c^	[106]
	*Momordica charantia*	quercetin-O-pentosylhexoside	HeLa, Hep2G, MCF-7, NCI-H469, A549	112–500	10,000	0.01	0.5 ^d^	[37]
	*Murraya paniculata,* Jack leaf	caryophyllene (20.93%).	L6, MIAPaCa2, PA1	13.1–55.2	4	3.3	13.9 ^b^	[107]
	*Nepeta* sp.	trans-caryophyllene (17.53%)	A549, C450, KB, **T-lymphocytes**	133.2–5267.9	2500	0.05	2.1 ^c^	[63]
	*Nephrolepis exaltata*	2,4-hexadien-1-ol (16.1%)	A-558, HCT-124, MCF16	52.5–97.4	125	0.4	0.8 ^d^	[108]
	*Ocimum basilicum*	linalool	HeLa, MCF-7, Hep3B	53.2–80.4	1	53.2	80.4 ^b^	[43]
	*Ocimum canum* Sims	thymol (42.15%)	Peritoneal macrophages, MCF7	63.9–315.3	100–1250	0.05	3.2 ^c^	[109]
	*Ocotea caniculata* sp.	α-pinene (9.8–22.5%)	MCF7	64	1250	0.05	0.05 ^d^	[110]
	*Opuntia macrorhiza*	camphor (39%)	HeLa, HepG8	206–359	450–1850	0.1	0.8 ^d^	[111]
	*Origanum vulgare*	carvacrol (2-meth yl-5-(1-methylethyl)phenol) (59.46%)	A549	14	6.3–25	0.6	2.2 ^c^	[112]
	*Pelargonium graveolens*	citronellol (27%)	HCT-15, HeLa, HepG2, MCF-7, NCI-H460	63.7–116.7	130	0.5	0.9 ^d^	[96]
	*Peucedanum dhana*	trans-piperitol (51.23%)	3T3L1, A549, Hela, SW480	10.2–961.4	62.5	0.2	15.4 ^b^	[113]
	*Pimenta dioica (All spices)*	eugenol (90%)	THP-3	29.6	500	0.06	0.1 ^d^	[72]
	*Piper nigrum* (Black pepper)	b-pinene (34.4%)	A549, FemX, HeLa, K562, LS-174, **MRC-5**	25.6–56.7	1250	0	0 ^d^	[92]
	*Pistacia khinjuk*	myrcene (16.5%)	DU-145, MCF-11, PC3, HeLa	29.6–169	150	0.2	3.4 ^c^	[35]
	*Polyalthia viridis* Craib	germacrene D (46%)	MCF7, A549, HepG2	56.7–68.4	200	0.3	0.4 ^d^	[114]
	*Rosmarinus officinalis*	1,8-cineol (47.6%)	**L929**	0.25	32	0	0 ^d^	[115]
	*Salvia* sp.	ekaempferol3-O-(6″ O-acetilglucoside)-7-O-rhamnoside (1.5–63%)	A461, L931, HCT-116, HT-30, MCF-8, MOLT-5, MCF-10, MOLT-7	7–389.2	17.1–5000	0	1.9 ^c^	[116,117,118]
	*Satureja hortensis*	carvacrol (48.51%)	**phoenx-eco**, THLE4, WI40	31.6–56.5	3000	0.01	0.05 ^d^	[119]
	*Satureja natatorium*	thymol (43%)	COLO-205, HeLa, MCF-7, HepG2	82–1090	6.3–12.5	7.1	148.8 ^a^	[40]
	*Stachys parviflora*	terpenyl acetate (23.6%)	A2780, B16F10, HCT	16.5–31	9.1	1.8	3.4 ^c^	[36]
	*Syzygium aromaticum*	eugenol (75.1%)	HT29	13.5	3.1	4.3	4.3 ^c^	[120]
	*Tetraclinis articulata*	α-pinene (36.5%)	RAW 264.7	577.32	2000	0.3	0.3 ^d^	[66]
	*Teucrium multicaule*	germacrene D (50%)	SF268	78.9	625	0.1	0.1 ^d^	[121]
	*Thymus* sp.	p-cymene (29.52%) [38], carvacrol (55.6%) [122]	HeLa, A375, A549, **MRC-5**, LS 174 T, MRC-10	0.2–30.3	12.5–160	0.02	0.2 ^d^	[38,122]
	*Zanthoxylum acanthopodium*	γ-gurjunene (51.1%)	SK-LU-1, MCF-7, HepG2	16–35.6	512	0.03	0.07 ^d^	[123]
	*Zingiber*	b-phellandrene (24.0%) [124], α-pinene (22.1%) [125]	**HaCaT**, A549, K562, PC-3, **MRC-5**, A552, PC-6	10.5–200	312.5–1280	0.03	0.5 ^d^	[124,125]
*Enterobacter cloacae*	*Coriandrum sativum*	linalool (69.6%)	HeLa, HepG2, MCF-7, NCI-H460, PLP2	67–140	690	0.1	0.2	[126]
	*Dennettia tripetala*	2-methyl phenyl formate (56.05%)	**RBC**	600	100	6	6	[127]
	*Opuntia macrorhiza*	camphor (39%)	HCT19, HeLa, HepG6	206–359	1850	0.1	0.2	[111]
	*Origanum minutiflorum*	carvacrol (81.5%)	HeLa, HepG2, MCF-7, NCI-H460, PLP2	77–151	690	0.1	0.2	[126]

**Bold:** normal cell lines; normal font: cancerous cell lines; SI_min_: minimum selectivity index; SI_max_: maximum selectivity index. (^a^) = maximum selectivity index > 100; (^b^) = maximum selectivity index 10–99; (^c^) = maximum selectivity index 1–9; (^d^) = maximum selectivity index < 1. Full names of cells are provided in Supplementary Materials.

3. Efficacy and safety of EOs used against *E. coli*.

*E. coli* is frequently used as a standard test organism in antimicrobial screening assays to evaluate the general antibacterial potential of new compounds before testing on more clinically relevant or MDR strains. This is because *E. coli* serves as a representative Gram-negative bacterium that is well-characterized, easily cultured, and widely available in microbial culture collections as well as having well-defined susceptibility or resistance profiles [128]. Hence, a larger number of in vitro studies focussed on *E. coli* compared to other bacterial species in this review.

The antimicrobial effectiveness and safety profiles of EOs tested against *E. coli* varied significantly across plant species. Based on their MIC and SI_max_ values, several Eos, including *Mentha* sp. (MIC 0.13–789.3 μg/mL; SI_max_ 845.4) [129,130], *S. nabateorum* (MIC 2.3 μg/mL; SI_max_ 484.4) [40] *Cinnamomum* sp. (MIC 0.49–6.3 μg/mL; SI_max_ 116.9) [88], and *Foeniculum vulgare* (MIC 128 μg/mL; SI_max_ 109.8) [131], and *Ocimum basilicum* (MIC 1 μg/mL; SI_max_ 80.3) [43], demonstrated good antibacterial activity against *E. coli* with wide therapeutic margins, indicating their strong potential for safe use as antimicrobial agents in clinical and industrial applications.

EOs from various *Mentha* sp. exhibited notable inhibitory activity against Gram-negative bacteria, particularly *E. coli*. These inhibitory effects are primarily attributed to their major constituents, such as menthol and menthone in peppermint (*M. piperita*), carvone in spearmint (*M. spicata*), and pulegone in pennyroyal (*M. pulegium*) [62,132].

EOs extracted from *Cinnamomum* sp., commonly used as a food spice, have been identified as highly effective antibacterial agents against several Gram-negative ESKAPEE pathogens. Notably, these oils exhibit strong inhibitory activity against *E. coli*, with reported MIC values ranging from 0.49 to 6.3 μg/mL (Table 3). Cinnamon extracts and their EOs rich in eucalyptol, cinnamaldehyde, and eugenol exhibit strong antimicrobial effects [88,89] through multiple mechanisms, including disruption of the cell membrane, alterations in lipid composition, inhibition of ATPase activity and cell division, and interference with motility and biofilm formation, as well as anti-quorum sensing actions [133].

*Ocimum basilicum* (sweet basil, holy basil) is a widely cultivated aromatic herb traditionally used in many cultures for its therapeutic properties and that has gained considerable interest in modern pharmacological research. Its EOs and extracts exhibit remarkable antibacterial activity, particularly against Gram-negative ESKAPEE pathogens such as *E. coli*, *P. aeruginosa*, and *K. pneumoniae*, with MIC values as low as 1 μg/mL [43] (Table 1, Table 2 and Table 3). In addition, *O. basilicum* demonstrates potent inhibitory effects against a range of Gram-positive ESKAPPE pathogens, including MRSA [23], highlighting its broad-spectrum antimicrobial potential.

**Table 3:** Inhibitory concentration (IC_50_), MIC and corresponding SI values for EOs (arranged alphabetically, A–Z) tested against *E. coli*.

Pathogens	Plant Name or EO	Major Chemical Constituent (Amount or Relative Amount)	Cell Lines Used in Toxicity Test	Mammalian Cell Inhibitory Concentration (IC_50_) μg/mL	MIC (μg/mL)	SI_min_	SI_max_	References
*E. coli*	*Achillea setacea*	borneol (32.97%)	**L929**	4.68	7.5	0.6	0.6 d	[134]
	*Actinidia arguta*	squalene (23.08%)	PC-3, HT-31, A551	6067–13,646	6250	1	2.2 c	[33]
	*Aeschynomene indica*	(*E*)-caryophyllene (17.3%)	HepG2, **LO2**, MCF-7	40.8–74.1	1.3	32.7	59.3 b	[83]
	*Agastache rugosa*	flower: pulegone (34.1%), leaves: p menthan-3-one (48.8%)	SGC-7901	800	50	16	16 b	[135]
	*Aloysia citriodora* Palau	*β*-spathulenol (15.61%)	MCF7, P815	6–34.7	8370	0	0 d	[136]
	*Aniba parviflora*	leaves: β-phellandrene (15.1%), branches: γ-eudesmol (16.8%)	MCF-8	67.9	19.5	3.5	3.5 c	[85]
	*Aquilaria crassna*	β-caryophyllene	**CCD-18Co, NIH/3T3-L1**, MCF-7, K562, PC3, ME-180, HT-29, PANC-1	27–612	9	3	68 b	[31]
	*Artemisia* sp.	β-thujone (89.54%) [41]; camphor (30.21%) [70]	HeLa, lymphocyte cells	7.1–5711	2.5–2500	0	1316.7 a	[41,70]
	*Asteraceae caerulescens*	eavana ether (17.3%)	MDA-MB 231, HCT116, A375	5.2–7.6	8	0.6	1 c	[74]
	*Bocageopsis multiflor*	cis-linalooloxide (furanoid) (33.1%)	**L929**	1.4	4.7	0.3	0.3 d	[86]
	*Brachychiton discolor*	leaves: a-farnesene (34.57%); flowers: n-heptacosane (29.5%)	HepG2, MCF-7, A-549, MCF-9, HepG4	8–30.5	1–31.5	0.3	31.1 b	[137]
	*Calothamnus quadrifidus*	1,8-cineole (53.98%)	Caco-2, HCT-116, A-549, HepG-2	3–6	1.3	2.4	4.7 c	[138]
	*Cedrus atlantica*	*α*-pinene (81.49%)	MCF-7	143.1	250	0.6	0.6 d	[139]
	*Cinnamomum* sp.	eucalyptol (65.87%)	MCF-7, HepG2, HCT-116, **adipose-derived mesenchymal stem cells**	9.1–83.5	0.49–6.3	13.4	116.9 a	[88]
	*Cistus* sp	α-pinene (42.1%)	PLP2, PC-3, MCF-7, NIH-3T3, SH-SY5Y, AGS, CaCo2, NCI-H460,	13.9–207.3	512–600	0.02	0.3 d	[39,90]
	*Cousinia* sp.	m-benzyl benzyl alcohol (46.7%)	T-47D, A549, A2780, Hep-G2,	4.5–32.2	62.5–125	0.04	0.5 d	[56]
	*Cryptocarya alba*	α-terpineol (24.96%)	HK-2, MCF-7, MCF10A	32–64	36	0.9	1.8 c	[140]
	*Cupressus sempervirens*	α-pinene (55.6%)	PLP2, CaCo2, MCF-7, NCI-H460	19.9–289.3	2500	0	0.1 d	[39]
	*Curcuma* sp. (Turmeric)	8,9-dehydro-9-formyl-cycloisolongifolene (2.37–42.59%	LNCaP, B16, HepG2	4–429.3	420.5–711	0	0.8 d	[141]
	*Dennettia tripetala*	2-methyl phenyl formate (56.05%)	**RBC**	620	200	3.1	3.1 c	[127]
	*Dictamnus angustifolius*	tetramethylenecyclobutane (42.07%)	B16, MCF7	15–57	225	0.1	0.3 d	[142]
	*Diospyros discolor*	(2Z,6E)-farnesol	J5, A549, HT-29	10.6–36.8	62.5	0.2	0.6 d	[48]
	*Dracocephalum kotschyi* Boiss	geranial (citral a) (12.1%)	HeLa, **lymphocyte** cells	26.4–4266.7	640	0	6.7 c	[97]
	*Eucalyptus globulus*	eudesmol (71.967%)	THP-1	3068–4057	1000	3	4.1 c	[143]
	*Ferula* sp.	δ-3-carene (72.6%) [99], caryophyllene oxide (33.9%) [100]	HCT-118, HCT-117, HT-30, HT-29, CEM/ADR5000, HCT-116, HT-29, U937, A549, HeLa	3.4–81	39–78	0	2 c	[99,100]
	*Ficus tikoua* Bur	palmitic acid (51.13%)	**MRC-9**, A553, PC-7, NCI-H1303, K566	31.7–161.8	6250	0	0 d	[101]
	*Filifolium sibiricum*	espatulenol (8.55%)	HepG-2, BGC-823, SKOV-3, MCF-7	780–3440	10,410	0.1	0.3 d	[144]
	*Foeniculum vulgare* Mill	trans-anethole (68.9%)	MCF-7	14,060	128	109.8	109.8 a	[131]
	*Gautheria procumbens*	methyl sali-cylate (99.96%)	**PBMCs**	58,340	6330	9.2	9.2 c	[145]
	*Gelsemium elegans*	α-terpineol (18.8%)	A-549, HepG2, MCF-7, HL-7702, HCT-116	60–160	320	0.2	0.5 d	[146]
	*Hedychium favum*/puerense	coronarin E (20.3%) [102], linalool (26.5%) [147]	**L929**, NCI-H1299, PC-3, K562, A549, **MRC-5**, RAW264.12, L934	72.9–370	9.8–6250	0	30.1 b	[102,147]
	*Heracleum* sp.	β-pinene (35.1%) [148], (Z)-falcarinol (80.0%) [149], (E)-nerolidol (28.5%) [150]	LS 174, Hela, A549,	5.9–58.8	0.08–8.6	0.7	311.9 a	[148,149,150]
	*Iberis amara*	4-carvomenthenol (18.75−22.13%),	HCT122, SW482	32.1	31.2	1	1 c	[151]
	*Illicium verum*	E-anethole 88.38%	MCF-7, 3T3, HeLa	57.3–131.7	100	0.6	1.3 c	[59]
	*Iris haynei*	diethyl phthalate (65.8%)	CaCo-2, HepG2, B16F1	757.9–915.2	25	30.3	36.6 b	[60]
	*Juniperus communis*	limonene (21.3%)	PLP2, CaCo2, AGS, NCI-H460, MCF-7	42–302.9	1250–2500	0.02	0.1 d	[39]
	*Laurelia sempervirens*	isazafrol (91.9%)	MCF-7, 786-0, ACHN	32–64	64	0.5	1 c	[140]
	*Laurus nobilis*	1,8-cineole (48.54%)	CaCo-2, MCF-7, B16F1	99.1–324.1	50	2	6.5 c	[61]
	*Limonium oleifolium*	γ-muurolene (10.8%)	J 775	90.2	3	30.1	30.1 b	[104]
	*Marrubium vulgare*	4aα,7α,7aα nepetalactone	PC-3, MDA-MB-231, MCF-7	30.1–135.6	100	0.3	1.4 c	[152]
	*Mentha* sp.	carvone (57.7–65.6%) [62,129], menthofuran (64.7–72%), (23–52.4%) [132]	MCF-7, **HUVEC**, A2780, A549, Caco-2, A549, **NIH 3T3**, T47D, MCF-8, HCT-117, LS-175, A550, MRC-6	36–975	0.13–789.3	0.6	845.4 a	[62,129,130,132]
	*Nepeta* sp.	4a,7,7a-nepetalactone (not reported)	PC-3, MDA-MB-231, MCF-7, MCF49, MCF38, MCF27, MCF16, **HUVEC**	30.9–85.8	22.5	1.4	3.8 c	[153]
	*Ocimum basilicum*	linalool	MCF-7, Hep3B	56.7–80.3	1	53.7	80.3 b	[43]
	*Ocimum canum* Sims	thymol (42.15%)	Peritoneal macrophages	315.3	200	1.3	1.3 c	[109]
	*Ocotea caniculata* sp.	α-pinene (9.8–22.5%)	MCF7	63.9	19.5	3.3	3.3 c	[110]
	*Opuntia microdasys*	camphor (40%)	Hela, HepG7, HCT20	206–359	950–1850	0.1	0.4 d	[111]
	*Origanum vulgare*	carvacrol (2-meth yl-5-(1-methylethyl)phenol) (59.46%)	A549	14	12.5	1.2	1.2 c	[112]
	*Peucedanum dhana A. Ham*	trans-piperitol (51.23%)	3T3L1, Hela, A549, SW480	10.2–961.4	62.5	0.2	15.4 b	[113]
	*Piper nigrum*	trans-β-caryophyllene (19.22%)	HEP-2, HeLa-2, PC-3, HepG2, MCF-7	5.2–22.1	2	2.7	11.3 b	[154]
	*Polyalthia viridis* Craib	germacrene D (46%)	A549, HepG2, MCF7	56.7–68.4	200	0.3	3.4 c	[114]
	Rose oil	citronellol (36%)	**Beas-2B**, A549	16.6–33.7	180	0.1	0.2 d	[49]
	*Rosmarinus officinalis*	1,8-cineole (26.4%)	HCT120	401.29	1000	0.4	0.4 d	[155]
	*Salvia* sp.	ekaempferol3-O-(6″O-acetilglucoside)-7-O-rhamnoside (1.5–63%)	**L929**, A460, HCT-116, MCF-7, MOLT-4, HT-29,	135.4–32,000	5000–64,000	0.03	2.2 c	[116,117,118]
	*Satureja hortensis*	carvacrol (48.51%)	**WI38**, **phoenx-eco**	31.6–56.5	4000	0	0.01 d	[119]
	*Satureja nabateorum*	thymol (43%)	MCF-7, COLO-205, HepG2, HeLa	82–1090	2.3	36.4	484.4 a	[40]
	*Stachys parviflora*	terpenyl acetate (23.6%)	B16F27, A2780, HCT	16.5–31	2.3	7.2	13.6 b	[36]
	*Syzygium aromaticum*	eugenol (75.1%)	HT29	13,510	6250	2.2	2.2 c	[120]
	*Telekia speciosa* (Flower)	roots: isoalantolacton (46.2%); flowers: nerol (11.9%); leaves: (E)-nerolidol (10.1%)	C32, **HaCaT**, A375	7.2–14.2	7800	0	0 d	[156]
	*Thymus* sp.	p-cymene (29.52%)	HepG-2, PC-3, LS 174 T, MRC-8, A552, HeLa, A375	0.2	1.6	0.1	0.2 d	[38]
	*Withania adpressa* Coss	caryophyllene (24.74%)	MCF-12	1000	51	19.6	19.6 b	[67]
	*Xylopia aethiopica*	pinocarvone (26.5%)	RAW 264.9	3.8	32	0.1	0.1 d	[68]
	*Zanthoxylum acanthopodium*	γ-gurjunene (51.1%)	SK-LU-1, MCF-7, HepG2, A-549	16–69.5	100–512	0.1	0.7 d	[123,157]
	*Zingiber*	b-phellandrene (24.0%)	PC-3, A549, K562, **MRC-5**, A553, PC-7	10.5–147.2	156.3–1560	0.1	1 c	[125,158]

**Bold** = normal cell lines; normal font = cancerous cell lines. SI_min_: minimum selectivity index; SI_max_: maximum selectivity index. (a) = maximum selectivity index > 100; (b) = maximum selectivity index 10–99; (c) = maximum selectivity index 1–9; (d) = maximum selectivity index < 1. Full names of cells are provided in Supplementary Materials.

**Figure 3:**
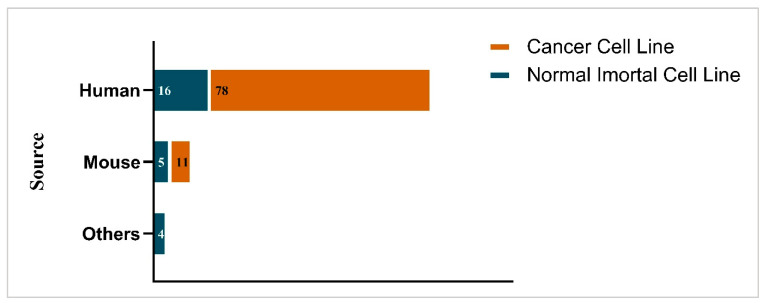
Trends in the types of cells used for cytotoxicity evaluation in the studies included in this review (normal versus cancer cells). Note: “Others” includes sheep blood and monkey blood cells.

Cell lines used in cytotoxicity assessment of EOs:

As shown in Figure 3, the cytotoxicity effects of EOs have been predominantly assessed using human-derived cancer cell lines, with 68% of the studies using these. In contrast, only a relatively small number of investigations have employed non-cancerous human cell lines, indicating limited evaluation of EO effects on more normal human cell models. Some studies also utilized mouse-derived cancerous and immortalized cell lines (Figure 3). Hence, comprehensive assessments of selectivity and potential toxicity toward normal cells are still limited, and this makes it difficult to draw conclusions about the safety and cytotoxicity of most EOs. Often, low SI values were attributable to the higher sensitivity of cancer cells compared to normal cells, as cancer cells typically exhibit altered metabolic pathways, compromised regulatory mechanisms, reduced protective mechanisms, and increased membrane permeability, making them more vulnerable to cytotoxic agents even at lower concentrations [159]. Growing evidence suggests that EOs display cancer-cell-selective cytotoxicity, characterized by lower IC_50_ values in cancer cells compared with healthy counterparts [160,161].

As examples, EOs from *Artemisia* species demonstrated pronounced cytotoxicity against human cervical carcinoma cells (HeLa) while remaining largely non-toxic to normal lymphocytes, reflecting an exceptionally high safety margin (max SI: 5711.7). At just 28 µg/mL, these oils inactivated 62.5% of HeLa cells, whereas a much higher concentration of 5600 µg/mL was needed to achieve 60.6% inactivation of lymphocytes [53]. Similarly, *Nepeta* species EOs showed minimal cytotoxicity toward healthy immune cells, with an IC_50_ of 5267.87 µg/mL, but they were markedly more toxic to cancer cell lines, achieving IC_50_ values below 430 µg/mL, even at the lowest tested concentrations [63]. *Dracocephalum kotschyi* Boiss further exemplifies this selectivity, exhibiting strong cytotoxicity toward HeLa cells (IC_50_ = 26.4 µg/mL) while sparing normal lymphocytes (IC_50_ = 4266.7 µg/mL) [97]. *Cinnamomum* extract shows potent and selective cytotoxicity toward major cancer cell lines (IC_50_ < 57.3 µg/mL for HCT-116, HepG2, and MCF-7) while exerting negligible toxicity on normal adipose-derived mesenchymal stem cells (AD-MSCs), which have an IC_50_ of 83,510 µg/mL [88,89]. Similarly, EOs from *Mentha* species combine effective antibacterial activity with strong selectivity for cancer cells. They exhibit IC_50_ values below 64 µg/mL for malignant cell lines, while their effect on normal HUVEC cells is much weaker (IC_50_ = 214 µg/mL), approximately four times higher than for cancer cells [62].

Many commonly studied spice- or herb-derived EOs (such as those from *Cuminum cyminum* (cumin; SI 0.1), *Curcuma* sp. (turmeric; SI 0.7), *Pimenta dioica* (allspice; SI 0.1), *Piper nigrum* (black pepper; SI 0.1), *Rosmarinus officinalis* (rosemary; SI 0), *Thymus* spp. (thyme; SI 0.2), and *Zingiber* spp. (ginger; SI 0.5)) have been tested against cancer cell lines (Table 2). Because normal cells were limited in these assessments (Figure 3), the reported SI values are often low, suggesting that antibacterial concentrations may overlap with cytotoxic thresholds. However, when normal cells are considered, many EOs exhibit far higher SI values, indicating a favorable safety margin.

These data underscore not only the need to carefully choose cell lines that are used for cytotoxicity testing but also highlight a potential use of EOs in the treatment of cancer cells. The reason for the selectivity towards cancer cells can be due to several factors, and these are highlighted in Table 4. These include elevated reaction oxygen species (ROS) in cancer cells that may lead to cell death responses, compromised antioxidant capacity, leaky mitochondria, and activation of signaling pathways.

It is possible that the over-reliance on the use of cancer-derived cells in toxicity studies is a reason why some EOs in clinical studies perform well, even though they have been shown in other studies to be toxic to cancer-derived cell lines. This is supported by a clinical study of 40 participants evaluating *Melaleuca alternifolia* (tea tree; 41% terpinen-4-ol), *Lavandula angustifolia* (lavender; 38% linalyl acetate), and *Eucalyptus globulus* (83% 1,8-cineole) for topical use over 90 days, in which single or combined application at appropriate doses was well tolerated, with no adverse effects reported [50]. Similarly, a randomized, double-blind, placebo-controlled clinical trial in 84 patients showed that the consumption of ginger was safe [162]. Furthermore, no adverse effects were reported in a study evaluating the safety of TTO and lavender oil exposure among 556 children aged 2–15 years, indicating that these oils are safe ingredients for use in personal care products formulated for children [163].

**Table 4:** Summary of possible reasons why cancer cells may be more prone to toxic effects of EOs.

Factor	Cancer Cells	Normal Cells
Basal ROS [164]	Elevated; near threshold	Lower; more margin to tolerate stress
Antioxidant capacity [165]	Often compromised	Robust (GSH, Trx, SOD systems)
Mitochondrial stability [166]	Leaky/dysregulated	Relatively intact
Signaling pathways [165]	Hyperactive (ERK/AKT/NF-κB/STAT3)	Balanced regulation
Cell cycle control [167]	Defective checkpoints	Functional checkpoints
Response to EOs [166]	ROS surge → apoptosis/autophagy/cell cycle arrest	Tolerable stress, minimal damage

GSH: Glutathione, Trx: Thioredoxin, SOD: Superoxide Dismutase, ERK: Extracellular-Signal-Regulated Kinase, AKT: Protein Kinase B, NF-κB: Nuclear Factor κB and STAT3: Signal Transducer and Activator of Transcription.

## 3. Methods

The current study follows a methodology consistent with a previous study [23]. Relevant papers were retrieved from electronic searches of PubMed, Medline, ScienceDirect, Scopus, Scientific Electron Library and Cochrane Library using the terms: “essential oils”, “plant extracts”, “safety”, “cytotoxicity”, “toxi*”, “antimicrobial”, “antibacterial”, “antibiotic”, “cell lines”, “human”, “animal”, “in-vitro”*,* covering publications from 2013–2024. These terms were used alone or in combination using Boolean operators (“AND”, “OR”, “NOT”). Only studies that reported both the MIC and the toxicity (which could be the hemolytic, cell-cidal or cell-growth-inhibitory concentrations) of EOs were included. Furthermore, the studies had to have evaluated the antimicrobial efficacy against the Gram-negative strains of the ESKAPEE pathogens *K. pneumonia*, *A. baumannii*, *P. aeruginosa*, *Enterobacter* sp., and *E. coli*. Studies that did not evaluate antimicrobial activity or that examined the effects of EOs on human physiology (including psychology and inflammatory responses) or their use in agriculture or food were excluded. The complete study selection process is presented in Figure 2.

Calculation of selectivity index:

The safety of EOs is commonly assessed using SI, which quantifies the balance between antimicrobial potency and cytotoxicity toward host cells. The SI is defined as the ratio of the 50% mammalian cell inhibitory concentration (IC_50_) or cytotoxicity concentration 50% (CC_50_) on mammalian cells to the minimum inhibitory concentration (MIC) against target microorganisms (e.g., SI = IC_50_/MIC). A higher SI indicates a greater therapeutic margin and lower toxicity to mammalian cells.

Operational definitions of safety of EOs based on SI can be divided into four categories:

A. Very high SI (maximum SI value ≥ 100): These EOs possess a wide safety margin, meaning that their MIC value is significantly lower than the cytotoxic dose. Such oils may be relatively safe for use under proper guidance and are less likely to cause adverse effects even when small dosing variations occur.
B. High SI (maximum SI value between 10 and 99): EOs within this range are also regarded as relatively safe, though they have a narrower margin of safety compared to those in the very high SI category. Careful dosage control may be important to prevent potential side effects. This has also been confirmed by previous studies, which state that EOs with an SI > 10 are more toxic to various bacteria and fungi with minimal harm to human cells.
C. Low SI (maximum SI value between 1–9): EOs with a low SI present a limited safety margin. The MIC dose is closer to the toxic dose, so even minor increases in concentration or exposure duration can result in harmful effects. These oils would require cautious handling and should probably be used under strict supervision, especially in clinical or therapeutic contexts.
D. Very low SI (maximum SI value < 1): EOs in this category are considered potentially hazardous, as their toxic dose is equal to or even lower than the MIC value. Such oils pose a significant risk of toxicity, and they are recommended not to be used. If used at all, they should be used with extreme caution, supported by strong clinical evidence and administered by qualified professionals.

This categorization has been used previously [23], and this approach has been adopted because the included studies encompassed multiple plant species and diverse cell types, resulting in SI values that varied markedly from very low to extremely high. While the majority of reported SI values fall within the 10–99 range, several studies reported SI values greater than 100, whereas others reported very low SI values (<1).

The calculated SI values from studies involving different concentrations, cell types, and plant species were ranked to define the minimum selectivity index (SI_min_) and maximum selectivity index (SI_max_), representing as the lowest and highest calculated ratios of cytotoxic to antimicrobial effective concentration observed for each EO across all tested cell lines and target microorganisms. For clinical applicability, EOs with SI_max_ > 10 are safe and promising alternatives to do further work, whereas an SI_max_ value < 9 indicates them being not selective to bacterial and toxic to mammalian cells.

## 4. Conclusions and Recommendations

To the authors’ knowledge, this is the first literature review to analyze EO safety using calculated SI values derived from Gram-negative ESKAPEE pathogens. The findings show that several EOs exhibit broad-spectrum antibacterial activity against Gram-negative ESKAPEE pathogens but demonstrate cytotoxic effects against diverse cancer cell lines while maintaining a high safety margin for normal cells. These characteristics warrant further investigation into their potential applications for treating infectious diseases and various types of cancer, with minimal impact on healthy cells.

However, because most EOs have been evaluated primarily on cancer cell lines and show SI values below 10, they may appear to pose safety risks. Nevertheless, these SI values are not definitive indicators of toxicity. Additional evidence from studies using normal cell lines and well-designed clinical investigations is needed to more accurately assess their safety profile.

## 5. Strength and Limitations of the Review

A key strength of this review is the classification of a wide range of EOs derived from food spices and traditional medicinal plants based on their safety profiles using calculated SI values. However, the study does not consider factors such as plant origin, extraction methods, or storage conditions, all of which can substantially alter the chemical composition of EOs. Such variations as well as bacterial strains and cytotoxicity assay types may subsequently affect their antibacterial activity, overall efficacy, and potential toxicity. Another limitation of this review is the use of a limited dataset comparing the cytotoxic effects of EOs on normal versus cancer cells.

Future directions:

Further comparative studies involving normal and cancer cell lines, together with clinical trials, are crucial for a more accurate assessment of the safety, selectivity, and potential risks of EOs.

## Data Availability

No new data were created or analyzed in this study. Data sharing is not applicable to this article.

## Supplementary Materials

The following supporting information can be downloaded at: https://www.mdpi.com/article/10.3390/antibiotics15030274/s1, Table S1: Full names of cells used for toxicity study.

## Abbreviations

AMR	Antimicrobial Resistance
EOs	Essential Oils
ESKAPEE	*Enterococcus faecium*, *Staphylococcus aureus*, *Klebsiella pneumoniae*, *Acinetobacter baumannii*, *Pseudomonas aeruginosa*, *Enterobacter* sp. and *Escherichia coli*.
GRAS	Generally Recognized as Safe
HMGECs	Human Meibomian Gland Epithelial Cells
IC_50_	50% Inhibitory Concentration
MDR	Multi-Drug Resistance
MIC	Minimum Inhibitory Concentration
MRSA	Methicillin-Resistant *S. aureus*
ROs	Reactive Oxygen Species
SI	Selectivity Indices
T4O	Terpinen-4-ol
TTO	Tea Tree Oil
WHO	World Health Organization
